# Emerging predominance of *Plasmodium vivax* and its contribution to severe malaria in Central Ethiopia: a mixed retrospective-prospective study from two tertiary hospitals

**DOI:** 10.1186/s12879-026-14383-3

**Published:** 2026-09-22

**Authors:** Ayto Addisu Negash, Mehreteab T. Woudineh, Kassim H. Mohammed, Eyob Z. Chafamo, Solomon Y. Tilahun, Biruk G. Awata, Abraraw E. Dires, Ashagre E. Hundeto, Etsegenet D. Dires, Adey A. Bogale, Tekiy M. Bedore

**Affiliations:** 1 Department of Emergency Medicine and Critical Care, Nigist Eleni Mohamed Memorial Comprehensive Specialized Hospital, Wachemo University, Hosanna, Ethiopia; 2 Department of Emergency Medicine and Critical Care, Worabe Comprehensive Specialized Hospital, Werabe University, Werabe, Ethiopia; 3 Department of Paediatric and Child Health, St. Paul’s Hospital Millennium Medical College, Addis Ababa, Ethiopia; 4 Department of Nursing, Nigist Eleni Mohamed Memorial Comprehensive Specialized Hospital, Wachemo University, Hosanna, Ethiopia; 5 Department of Internal Medicine, Nigist Eleni Mohamed Memorial Comprehensive Specialized Hospital, Wachemo University, Hosanna, Ethiopia; 6 Department of Family Medicine, St. Paul’s Hospital Millennium Medical College, Addis Ababa, Ethiopia; 7 Department of Emergency Medicine and Critical Care, St. Paul’s Hospital Millennium Medical College, Addis Ababa, Ethiopia

**Keywords:** Malaria, *Plasmodium vivax*, Severe malaria, Species shift, Ethiopia, Emergency department

## Abstract

**Background:**

Malaria remains a major public health challenge in Ethiopia. While Plasmodium falciparum has historically been associated with severe disease, the clinical importance of Plasmodium vivax is increasingly recognized.

**Objective:**

This study aimed to determine the prevalence, species distribution, severity, associated factors, and clinical outcomes of malaria among adults attending emergency departments in Central Ethiopia.

**Methods:**

A mixed retrospective-prospective observational study was conducted in the adult emergency departments of two tertiary hospitals from October 1 to December 31, 2025. Severe malaria was defined according to the 2014 World Health Organization (WHO) criteria. The association between malaria species and severe malaria was examined using bivariate logistic regression.

**Results:**

Among 318 laboratory-confirmed malaria cases, P. vivax was the predominant species (49.7%), followed by P. falciparum (38.4%) and mixed infections (11.9%). Severe malaria was diagnosed in 65.7% of patients, with severe anaemia (haemoglobin < 5 g/dL) being the most common severity criterion (33.3%). Severe malaria occurred in 65.2% of patients with P. vivax and 67.2% with P. falciparum. No statistically significant association was found between malaria species and severe malaria. Most patients (62.3%) were discharged from the emergency department, while 27.7% were admitted to medical wards. In-hospital mortality was 4.4%.

**Conclusion:**

P. vivax was the predominant malaria species among adults presenting to the two tertiary hospitals, and both P. vivax and P. falciparum were associated with substantial severe disease. Severe anaemia was the most common manifestation. Strengthening early diagnosis, prompt treatment, species-specific case management, and appropriate radical cure for P. vivax remains important for malaria control and elimination efforts.

**Clinical trial statement:**

This manuscript does not describe a clinical trial.

## Introduction

### Background

Malaria remains one of the major infectious diseases affecting sub-Saharan Africa. In 2024, an estimated 282 million malaria cases and 610,000 malaria-related deaths occurred globally, with the WHO African Region accounting for approximately 94% of malaria cases and 95% of malaria deaths. This disproportionate burden highlights the continuing importance of malaria surveillance, prevention, and case management in African countries [1].

Ethiopia has made substantial progress in malaria prevention and control over the past two decades; however, malaria transmission remains highly heterogeneous and has recently shown a marked resurgence. Approximately 75% of the country’s landmass is considered malaria-endemic, and around 69% of the population residing in these areas is at risk of infection [2]. Following a substantial decline in malaria burden and approximately 900,000 reported cases in 2019, Ethiopia experienced a resurgence beginning in late 2021, with epidemics reported across most regions from 2022 onward [2]. Between January and October 2024, more than 7.3 million malaria cases and 1,157 deaths were reported, representing the highest annual number of cases recorded during the preceding seven years. Notably, *Plasmodium falciparum* accounted for more than two-thirds of reported cases in 2024, while the geographic extent of endemic areas also increased compared with previous years [2]. These recent changes indicate that malaria epidemiology in Ethiopia is dynamic and continues to require geographically specific surveillance and updated clinical evidence.

Malaria transmission in Ethiopia is predominantly caused by *Plasmodium falciparum* and *Plasmodium vivax*, which coexist in many endemic areas but show considerable geographic and temporal variation in their relative contribution [2–4]. Historically, *P. falciparum* has been the predominant species and has been associated with a large proportion of severe malaria and malaria-related mortality, whereas *P. vivax* has remained an important cause of malaria, particularly in areas characterized by unstable or lower-intensity transmission. A systematic review and meta-analysis of Ethiopian studies conducted between 2000 and 2020 identified *P. falciparum* in 66.6% and *P. vivax* in 32.7% of confirmed malaria infections, demonstrating the substantial contribution of both species and considerable regional variation in *P. vivax* prevalence [3]. More recent Ethiopian evidence continues to demonstrate substantial co-circulation of the two species, although their relative proportions vary considerably between regions and over time [4–6].

Importantly, recent surveillance data suggest that the contribution of *P. vivax* has increased in several Ethiopian settings during the recent malaria resurgence. In the Amhara Region, analysis of 11 years of surveillance data from 2014 to 2024 demonstrated a marked resurgence of malaria, with annual cases increasing from approximately 248,000 in 2018 to 1.88 million in 2024. During the same period, *P. vivax* incidence increased approximately 11-fold, from 2.7 per 1,000 population in 2018 to 32.4 per 1,000 in 2024, while the proportion of malaria cases attributable to *P. vivax* increased from 24.9% to 43% [5]. Similarly, a five-year surveillance study from Mattu Karl Specialized Comprehensive Hospital in southwestern Ethiopia found that *P. vivax* accounted for 49.1% of confirmed malaria cases between 2020 and 2024, compared with 37.8% for *P. falciparum* and 13.1% for mixed infections; the overall malaria positivity rate increased from 9.2% in 2020 to 17.7% in 2024 [6]. These findings demonstrate that although *P. falciparum* remains the predominant malaria species nationally, *P. vivax* is increasingly important in several regional settings and that species distribution is highly heterogeneous across Ethiopia.

Evidence from Central Ethiopia further emphasizes the importance of monitoring this changing epidemiology. A recent analysis of malaria surveillance data from Hadia, Gurage, and Yem zones covering 2019–2024 identified 86,604 confirmed malaria infections, of which 61% were attributed to *P. falciparum* and 39% to *P. vivax*. The study also demonstrated an overall increasing trend in malaria infections during the study period, with significant variation in species distribution across districts, seasons, and years [7]. Such findings indicate that national estimates alone may not adequately describe local malaria epidemiology and support the need for contemporary, setting-specific clinical and epidemiological studies.

Although *P. vivax* was historically regarded as relatively benign compared with *P. falciparum*, accumulating evidence has demonstrated that vivax malaria can cause severe and potentially life-threatening disease. Severe *P. vivax* malaria has been associated with severe anaemia, thrombocytopenia, respiratory distress, hypoglycaemia, hepatic and renal dysfunction, shock, and other organ complications [8, 9]. In Ethiopia, a clinical study conducted in Mendi, northwestern Ethiopia, found that 14.3% of patients with *P. vivax* mono-infection experienced symptoms meeting criteria for complicated or severe malaria, including severe anaemia, prostration, respiratory distress, persistent vomiting, hyperpyrexia, and hypoglycaemia [10]. More recent clinical literature continues to emphasize that severe vivax malaria is an important, although incompletely characterized, clinical problem and that the burden and manifestations of severe disease vary substantially between endemic settings.

The recent resurgence of malaria in Ethiopia, together with increasing regional contributions of *P. vivax*, substantial spatial and temporal variation in species distribution, and growing recognition of severe *P. vivax* disease, underscores the need for updated clinical and epidemiological evidence. In Central Ethiopia, where *P. falciparum* and *P. vivax* are co-endemic, contemporary hospital-based data are particularly important for characterizing species-specific malaria patterns and the clinical spectrum of disease. Such evidence can contribute to improved diagnosis and case management, strengthen surveillance of changing malaria epidemiology, and inform targeted malaria control and elimination strategies.

### Objectives

The general objective of this study was to determine the prevalence, species distribution, severity, associated factors, and clinical outcomes of malaria among adult patients attending emergency departments in two tertiary hospitals in Central Ethiopia, with particular emphasis on the emerging role of *P. vivax* in severe disease. The specific objectives were:

1. To determine the prevalence and species distribution of malaria.
2. To describe the socio-demographic and clinical characteristics of patients with malaria.
3. To assess severity patterns and complications according to WHO criteria.
4. To compare severity and outcomes between *P. vivax* and *P. falciparum* infections.
5. To examine the association between malaria species and severe malaria.

## Materials and methods

### Study design

This study employed a mixed retrospective–prospective observational design. Of the 318 laboratory-confirmed malaria cases included in the study, 232 (73.0%) were enrolled prospectively with real-time collection of clinical, laboratory, management, and outcome data, while 86 (27.0%) were identified retrospectively through review of existing hospital medical records. Both components covered the predefined study period and were combined to include all eligible cases and assess malaria species distribution, clinical characteristics, severity, management, and outcomes.

### Study area and period

The study was conducted in the adult emergency departments of Nigist Eleni Mohamed Memorial Comprehensive Specialized Hospital (Wachemo University, Hosanna) and Werabe Comprehensive Specialized Hospital (Werabe University, Werabe), Central Ethiopia. Both are tertiary referral hospitals serving urban and rural populations from surrounding areas with varying malaria transmission intensities. The study was conducted from October 1 to December 31, 2025.

### Study population and eligibility criteria

The study population comprised patients aged ≥ 15 years who presented to the emergency departments of the two participating hospitals during the study period and had laboratory-confirmed malaria by microscopy or a malaria rapid diagnostic test (RDT). Patients with incomplete medical records or missing key eligibility or study data were excluded. Patients aged < 15 years were not included.

### Sampling and sample size

All available eligible cases identified during the predefined three-month study period were included. A total of 341 adult malaria patients were initially screened across the two participating hospitals. Of these, 23 patients were excluded because of variable-specific incomplete medical records or missing key study data, resulting in a final cohort of 318 cases, comprising 232 prospective and 86 retrospective cases.

No formal sample size or power calculation was performed because the study used a time-bound convenience sampling approach and aimed to include all available eligible cases during the predefined study period. Thus, the final sample size was determined by the number of eligible cases available within the study timeframe rather than by a prespecified statistically powered sample.

### Data collection

Data were collected using a standardized, pre-tested data-collection tool adapted from relevant WHO malaria surveillance guidelines. The tool captured sociodemographic characteristics, presenting clinical features, laboratory findings, malaria species, severity indicators, emergency department length of stay, disposition, treatment, and final hospital outcomes. Laboratory variables included haemoglobin, platelet count, creatinine, and parasite density where available.

For prospectively enrolled patients, data were collected in real time by trained research assistants using structured case-report forms. For retrospectively identified patients, relevant information was extracted from existing patient medical records, laboratory registers, and emergency department registers. All data collectors received training on the study protocol and data-collection procedures before commencement of data collection.

### Operational definitions and severity classification

Severe malaria was classified according to the 2014 World Health Organization (WHO) criteria for severe malaria, based on the clinical and laboratory findings available for each patient. The applicable WHO diagnostic thresholds were applied where the corresponding clinical or laboratory data were available.

Hyper parasitaemia was defined as a parasite density > 10%. Quantitative parasite-density data were available for 45 patients (14.2%), all of whom were enrolled prospectively; therefore, the hyper parasitaemia criterion could not be assessed in the retrospective cohort. Similarly, although serum bilirubin measurements were available where documented, the parasite-density component of the WHO criterion for severe *P. falciparum* malaria with jaundice could not be assessed in patients without quantitative parasite-density data.

### Data quality control

To ensure data quality, research assistants received training and were supervised regularly by the principal investigator. Before the main data abstraction, the data abstraction tool was pre-tested on 10% of the eligible records to assess its clarity, completeness, and applicability. Records used for the pre-test were excluded from the final analysis and were not included in the analytical dataset. Double data entry was performed for a random sample of 20% of the cases, and discrepancies were reviewed and resolved by senior investigators. Regular checks for completeness, consistency, and accuracy were conducted throughout the data-collection and data-entry processes.

### Data processing and statistical analysis

Data were entered into and analysed using IBM SPSS Statistics version 27.0. Data were cleaned and checked for completeness and consistency before analysis. Descriptive statistics were used to summarize the study population and clinical characteristics. Categorical variables were presented as frequencies and percentages, while continuous variables were summarized using means with standard deviations or medians with interquartile ranges, as appropriate. Due to substantial missing data for most potential predictor variables, formal multivariable logistic regression incorporating multiple covariates was not feasible. The primary analysis therefore examined the association between malaria species and WHO-defined severe malaria using bivariate logistic regression. A two-sided p-value < 0.05 was considered statistically significant.

### Ethical declaration

Ethical approval for the study was obtained from the Medical Ethical and Research Ethics Evaluation Committee of Nigist Eleni Mohamed Memorial Comprehensive Specialized Hospital (Protocol No. NEMMCSH/001/2025), with full approval granted for the study period. The study was conducted in accordance with applicable institutional and national ethical requirements and the principles of the Declaration of Helsinki.

Consent to Participate: Written informed consent was obtained from patients enrolled prospectively, in accordance with the approved study protocol. For patients included retrospectively through review of existing medical records, data were obtained from routinely documented hospital records in accordance with the approved protocol. All study data were anonymized and handled confidentially.

Consent for publication: Permission to conduct and publish the study findings was obtained from the respective hospital administrations.

## Result

**Fig. 1:**
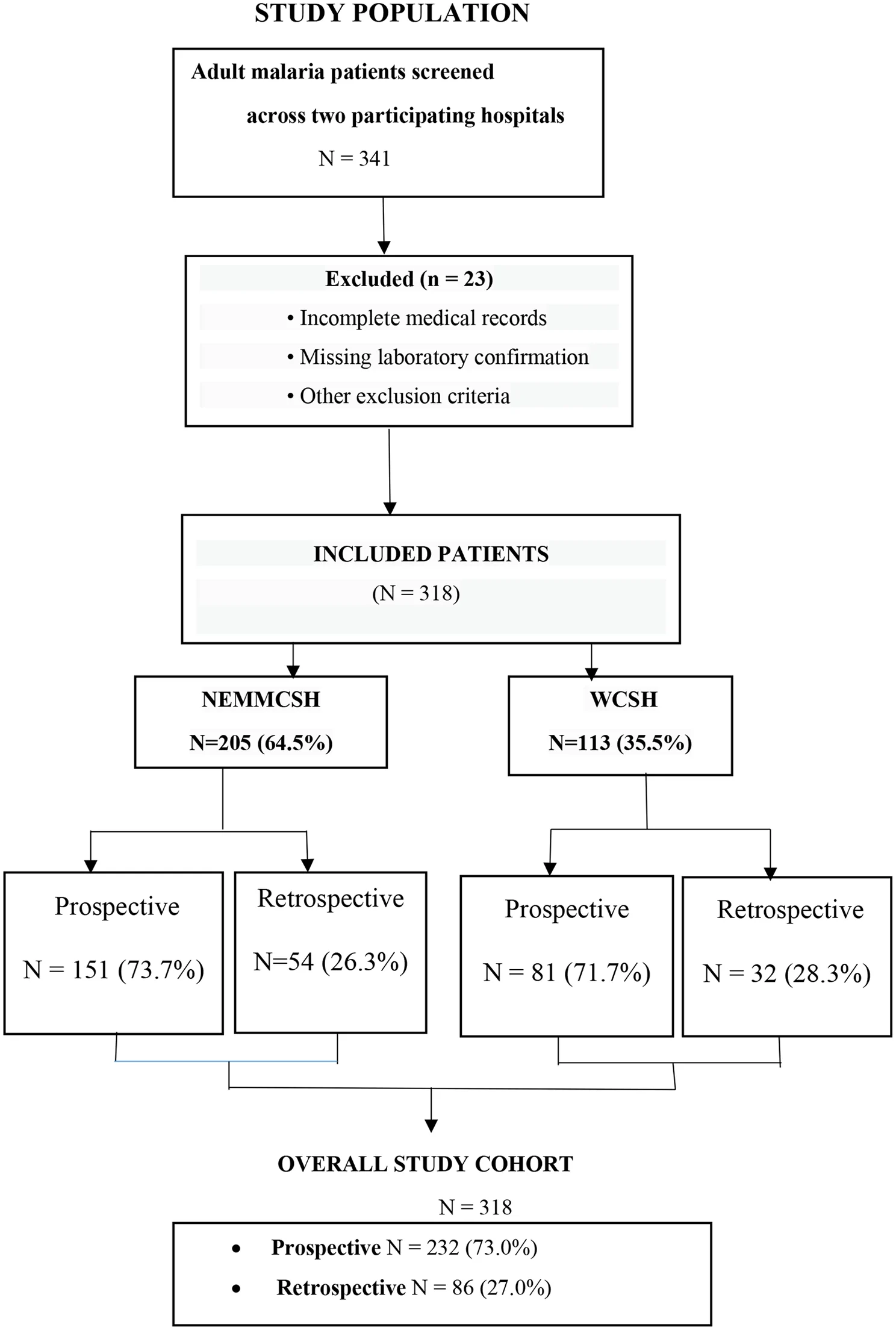
Flow diagram of study participants in the mixed retrospective–prospective study of malaria at NEMMCSH and Werabe Comprehensive Specialized Hospital, Central Ethiopia, October–December 2025

During the three-month study period (October–December 2025), 341 adult malaria patients were initially screened across the two participating tertiary hospitals. After excluding 23 patients because of incomplete medical records, missing key study data, or other exclusion criteria, 318 patients were included in the final cohort. Of these, 205 (64.5%) were from NEMMCSH and 113 (35.5%) were from Werabe Comprehensive Specialized Hospital (WCSH). At NEMMCSH, 151 patients were enrolled prospectively and 54 retrospectively, whereas at WCSH, 81 were enrolled prospectively and 32 retrospectively. Overall, 232 (73.0%) patients were enrolled prospectively and 86 (27.0%) retrospectively (Fig. 1).

### Socio-demographic characteristics

Of the 318 participants, 162 (50.9%) were male, and 156 (49.1%) were female. The majority (202, 63.5%) were aged 15–35 years. Among the 156 female participants, 36 were pregnant (27.7% of women with recorded pregnancy status; 11.3% of the total cohort). Pregnancy status was not recorded in 26 women. Rural residents accounted for 173 cases (54.4%). Most patients (205, 64.5%) presented to Nigist Eleni Mohamed Memorial Comprehensive Specialized Hospital (Table 1).

**Table 1:** Socio-demographic characteristics of malaria patients attending emergency departments at two tertiary hospitals in Central Ethiopia, October–December 2025 (*N* = 318)

S. No	Variables	Category	Number	Percent (%)
1	Age of participants	15–35	202	63.5
		36–55	96	30.2
		≥ 56	20	6.3
2	Sex	Male	162	50.9
		Female	156	49.1
3	Pregnancy status	Pregnant	36	11.3
		Not pregnant	94	29.6
		Not recorded	26	8.2
4	Place of residence	Urban	145	45.6
		Rural	173	54.4
5	Name of Facility	Werabe	113	35.5
		NEMMCSH	205	64.5

### Malaria diagnostic methods and species distribution

Microscopy was used in 273 (85.8%) cases and rapid diagnostic tests (RDT) in 45 (14.2%) cases. *P. vivax* was the predominant species, accounting for 158 (49.7%) cases, followed by *P. falciparum* with 122 (38.4%) cases and mixed infections with 38 (11.9%) cases. The *P. vivax* to *P. falciparum* ratio among the study participants was approximately 1.30:1 (Table 2A). Parasite density was recorded in only 45 (14.2%) cases, all prospectively enrolled, and of which 20 (6.3% of total) had hyper parasitaemia (≥ 10%) (Table 2B).

Table 2A and 2B. Malaria diagnostic methods and species distribution among patients attending emergency departments in Central Ethiopia, October–December 2025 (*N* = 318).

**Table 2A:** Diagnostic method and malaria species distribution

S. No	Variables	Category	Number	Percent
1	Malaria diagnostic method	Microscopy	273	85.8
		RDT	45	14.2
2	Malaria Species	*P. falciparum*	122	38.4
		*P. vivax*	158	49.7
		Mixed	38	11.9

**Table 2B:** Parasite density distribution among malaria patients with recorded parasitaemia

Parasite Density	Number	Percent of those with recorded density (%)	Percent of total patients (*N* = 318) (%)
< 10% parasite density	25	55.6	7.9
≥ 10% parasite density (Hyper parasitaemia)	20	44.4	6.3
**Total with recorded density**	**45**	**100**	**14.2**
No record / Not done	273	—	85.8

### Clinical manifestations

The most common presenting symptoms were fever (243, 76.4%), chills/rigors (197, 61.9%), headache (131, 41.2%), prostration (130, 40.9%), and shortness of breath (105, 33.0%). Altered mental status was observed in 61 (19.2%) patients and convulsions in 25 (7.9%) (Table 3).

**Table 3:** Clinical manifestations among malaria patients attending emergency departments in Central Ethiopia, October–December 2025 (*N* = 318)

S. No	Variables	Category	Number	Percent
1	Fever	Yes	243	76.4
		No	73	23.0
		Not Recorded	2	0.6
2	Chills/Rigors	Yes	197	61.9
		No	120	37.7
		Not Recorded	1	0.3
3	Headache	Yes	131	41.2
		No	187	58.8
4	Vomiting	Yes	87	27.4
		No	230	72.3
		Not Recorded	1	0.3
5	Prostration	Yes	130	40.9
		No	184	57.9
		Not Recorded	4	1.3
6	Altered Mental Status	Yes	61	19.2
		No	255	80.2
		Not Recorded	2	0.6
7	Convulsion	Yes	25	7.9
		No	290	91.2
		Not Recorded	3	0.9
8	Shortness of breath	Yes	105	33.0
		No	206	64.8
		Not Recorded	7	2.2
9	Jaundice	Yes	11	3.5
		No	260	81.8
		Not Recorded	56	17.6
10	Bleeding	Yes	7	2.2
		No	253	79.6
		Not Recorded	58	18.2

### Haematological and laboratory findings

The mean haemoglobin level was 9.56 g/dL. Severe anaemia (Hb < 5 g/dL) was present in 106 (33.3%) patients and occurred at similar rates in *P. vivax* (32.3%) and *P. falciparum* (34.4%) infections. Severe thrombocytopenia was significantly more frequent in *P. vivax* infections (28.5%) than in *P. falciparum* infections (9.8%). Abnormal creatinine levels were recorded in 48 (15.1%) patients.

### Malaria severity

Overall, 209 (65.7%) patients fulfilled WHO criteria for severe malaria. When stratified by malaria species, severe malaria was observed in 82 (67.2%) of the 122 patients with *P. falciparum*, 103 (65.2%) of the 158 patients with *P. vivax*, and 24 (63.2%) of the 38 patients with mixed infections. Thus, the proportion of severe malaria was comparable across the three infection groups, with *P. falciparum* having the highest proportion of severe cases (Table 4).

**Table 4:** Distribution of malaria species according to WHO severity classification among patients in Central Ethiopia, October–December 2025 (*N* = 318)

WHO severity classification	*P. falciparum*, *n* (%)	*P. vivax*, *n* (%)	Mixed infection, *n* (%)	Total, *n* (%)
Uncomplicated malaria	40 (32.8)	55 (34.8)	14 (36.8)	109 (34.3)
Severe malaria (≥ 1 WHO criterion present)	82 (67.2)	103 (65.2)	24 (63.2)	209 (65.7)
Total	122 (100.0)	158 (100.0)	38 (100.0)	318 (100.0

The most common severity indicators were severe anaemia (33.3%) and impaired consciousness (19.2%). Other complications included acute kidney injury (6.9%), hyper parasitaemia (6.3%), and pulmonary oedema/ARDS (3.5%) (Table 5).

**Table 5:** Distribution of WHO malaria severity indicators among patients in Central Ethiopia, October–December 2025 (*N* = 318)

S. No	Variables	Category	Number	Percent (%)
1	Impaired consciousness	Yes	61	19.2
		No	251	80.8
2	≥ 2 convulsions	Yes	24	7.5
		No	294	92.5
3	Severe anaemia (Hgb < 5 g/dL)	Yes	106	33.3
		No	212	66.7
4	Abnormal bleeding/DIC	Yes	8	2.5
		No	310	97.5
5	Hypoglycaemia (plasma glucose < 40 mg/dL])	Yes	6	1.9
		No	312	98.1
6	Metabolic acidosis (clinical)	Yes	2	0.6
		No	316	99.4
7	Renal impairment (serum creatinine > 3 mg/dL or urea > 20 mmol/L)	Yes	22	6.9
		No	294	92.5
		Not recorded	2	0.6
8	Jaundice (serum bilirubin > 3 mg/dL)	Yes	11	3.5
		No	307	96.5
9	Pulmonary edema/ARDS (clinical/radiological evidence)	Yes	11	3.5
		No	307	96.5
10	Shock	Yes	4	1.3
		No	314	98.7
11	Hyper parasitaemia (parasite density > 10%)	Yes	20	6.3
		No/Not record	298	93.7
12	WHO severity (≥ 1 WHO criteria present)	Uncomplicated malaria	109	34.3
		Severe malaria	209	65.7

### Patient outcomes and disposition

The median length of stay in the emergency department was 8–12 h. Most patients (198, 62.3%) were discharged directly from the ED, 88 (27.7%) were admitted to the ward, and 16 (5.0%) required ICU/HDU care. The in-hospital mortality rate was 14 (4.4%) (Table 6).

**Table 6:** Patient outcomes and disposition

Variable / Category	Total (*N* = 318)	Percent (%)
**1. ED Length of Stay**		
< 4 h	1	0.3%
4–8 h	122	38.4%
8–12 h	43	13.5%
12–24 h	64	20.1%
> 24 h	88	27.7%
**2. ED Disposition**		
Discharged home from the ED	198	62.3%
Admitted to the ward	88	27.7%
Admitted to ICU/HDU	16	5.0%
Transfer to another facility	6	1.9%
Left against medical advice	6	1.9%
Death	4	1.3%
**3. Outcome Type (Final Hospital Outcome)**		
Discharged alive (Recovered)	291	91.5%
Died	14	4.4%
Transferred to another facility	5	1.6%
Left against medical advice	8	1.9%

### Association of species type with severe malaria

No statistically significant association was found between malaria species and WHO-defined severe malaria.

## Discussion

This mixed retrospective–prospective study conducted in the emergency departments of two tertiary hospitals in Central Ethiopia found that *Plasmodium vivax* was the predominant malaria species, accounting for 49.7% of confirmed cases, compared with 38.4% for *P. falciparum* and 11.9% for mixed infections. This finding is consistent with recent reports from different parts of Ethiopia showing a substantial and, in some settings, increasing contribution of *P. vivax* to the malaria burden [3–12]. The predominance of *P. vivax* observed in the present study highlights the importance of considering both major malaria species in local surveillance and case-management strategies.

The study population was predominantly composed of young adults, with more than half residing in rural areas. Clinically, 65.7% of patients fulfilled WHO criteria for severe malaria, with severe anemia being the most frequent severity criterion. The proportion of severe malaria was comparable between *P. vivax* and *P. falciparum* infections, occurring in 65.2% and 67.2% of patients, respectively. Severe thrombocytopenia was more frequently observed among patients with *P. vivax* than among those with *P. falciparum*. These findings add to the growing evidence that *P. vivax* should not be regarded as uniformly benign and that it can be associated with severe clinical manifestations [13–17].

Several clinical and laboratory abnormalities were observed among patients with severe malaria, including severe anemia, impaired consciousness, renal impairment, jaundice, pulmonary oedema/ARDS, and hyper parasitaemia. Severe anemia was the most common WHO severity criterion. However, the observational design of the study does not permit causal inference regarding the mechanisms underlying severe disease. Furthermore, quantitative parasite-density data were available for only a small proportion of participants; therefore, findings involving hyper parasitaemia and other parasite-density-dependent criteria should be interpreted cautiously.

No statistically significant association was found between malaria species and WHO-defined severe malaria. This suggests that, within this hospital-based cohort, severe malaria was not restricted to a particular Plasmodium species. The finding should be interpreted cautiously in light of the limited variables available for analysis due to missing data, the relatively small sample size, the short study period, and the tertiary-care setting.

The high proportion of severe malaria observed in this study (65.7%) may partly reflect the tertiary-care setting of the participating hospitals, where patients with more complicated illness are preferentially referred from lower-level health facilities. Most patients (62.3%) were discharged directly from the emergency department, while 27.7% required ward admission and 5.0% required ICU/HDU care. The in-hospital mortality rate was 4.4%. These findings demonstrate that substantial morbidity and mortality remain associated with malaria among adults presenting to tertiary emergency departments, despite the availability of hospital-based management.

The substantial proportion of *P. vivax* among patients with severe malaria is consistent with previous reports from Ethiopia and other endemic settings [18, 19]. *P. vivax* has been associated with severe anemia, thrombocytopenia, respiratory complications, and organ dysfunction in previous studies. The present findings further support the need for continued clinical recognition of potentially severe *P. vivax* infection and appropriate assessment for WHO-defined severity criteria.

From a treatment and malaria-control perspective, the findings reinforce the importance of accurate species identification and prompt initiation of appropriate antimalarial therapy. According to the Ethiopian National Malaria Guidelines, treatment of uncomplicated *P. vivax* includes appropriate blood-stage therapy together with primaquine for radical cure when indicated and when the patient is eligible. Severe malaria requires urgent parenteral treatment and supportive care. Ensuring appropriate radical cure, including consideration of G6PD status and monitoring for potential hemolysis associated with primaquine, is important for reducing recurrent *P. vivax* infection. Strengthening diagnosis, treatment, follow-up, and surveillance for both *P. vivax* and *P. falciparum* will therefore remain important for malaria control and elimination efforts in Central Ethiopia.

### Limitations

This study has several limitations that should be considered when interpreting the findings. First, the mixed retrospective–prospective design may have introduced differences in the completeness, quality, and consistency of information between the two study components. Although 73.0% of participants were enrolled prospectively with real-time data collection, the retrospective component relied on information documented in existing medical records and was therefore more susceptible to missing, incomplete, or inconsistently recorded clinical and laboratory information. This may have introduced information bias and limited the ability to fully characterize some clinical and laboratory features.

Second, the study employed a time-bound convenience sampling approach over a three-month period, and no formal sample size or power calculation was performed. Consequently, the sample size was determined by the number of eligible patients presenting to the participating facilities during the study period, which may have limited the statistical power to detect relatively small or moderate associations. The study was also conducted in the emergency departments of only two tertiary hospitals. These facilities may preferentially receive patients with more severe or complicated illness, as well as patients referred from other health facilities, potentially resulting in referral or selection bias. Therefore, the findings may not be directly generalizable to patients managed at primary healthcare facilities, community settings, or other hospitals with different patient populations and referral patterns.

Third, quantitative parasite-density data were available for only 45 of the 318 participants (14.2%), all of whom were enrolled prospectively; no quantitative parasite-density measurements were available for the retrospective cohort. Consequently, hyper parasitaemia, defined as a parasite density > 10%, could not be assessed consistently across the entire study population. Similarly, although serum bilirubin measurements were available when documented, the absence of parasite-density data in the retrospective cohort limited comprehensive assessment of the parasite-density component of WHO criteria for severe *P. falciparum* malaria with jaundice. Accordingly, findings related to hyper parasitaemia and other severity criteria that depend on quantitative parasite-density measurements should be interpreted cautiously.

Fourth, several clinical and laboratory variables, including some treatment-related information, were incompletely documented, particularly among retrospectively identified patients. This may have resulted in information bias and limited assessment of the relationships between specific clinical characteristics, treatment practices, and outcomes. In addition, because the study included adults only, the findings cannot be extrapolated to children, who may have different clinical presentations, risk factors, disease severity patterns, and treatment outcomes. The study also did not assess long-term outcomes, post-discharge complications, or recurrent *P. vivax* infection and relapse, as these outcomes were beyond the scope and follow-up period of the study.

Fifth, the study did not systematically collect information on geographical accessibility, travel distance or travel time to healthcare facilities, migration status, previous healthcare utilization, or membership of specific ethnic or tribal populations. Consequently, the potential contribution of healthcare access, population mobility, and population-specific socioeconomic or demographic factors to malaria presentation and severity could not be adequately evaluated. These factors may be particularly relevant in understanding delays in accessing appropriate care and differences in malaria burden across populations and geographic areas.

Sixth, the relatively short study period, from October to December 2025, limits the ability to assess temporal and seasonal variation in malaria transmission, species distribution, clinical presentation, and disease severity. The findings therefore represent the epidemiological and clinical characteristics observed during the study period and should not be interpreted as describing the complete annual malaria pattern in the study area. Longer-term surveillance across multiple transmission seasons would be required to characterize seasonal variation and provide more representative estimates of malaria epidemiology and severity.

Finally, the observational nature of the study limits causal inference. Associations observed between patient characteristics and clinical outcomes should therefore not be interpreted as evidence of causation. Residual confounding by factors that were not measured or incompletely documented may also have influenced the observed relationships. In addition, the absence of a statistically significant association for particular variables should be interpreted cautiously, particularly where sample sizes were limited or relevant measurements were incompletely available.

Despite these limitations, this study provides important clinical and epidemiological data on malaria presentation, severity, management, and outcomes among adults presenting to two tertiary hospitals in Central Ethiopia. The findings contribute to the limited evidence from this setting and may help inform clinical practice and future research. Larger, multicentre studies using prospective sampling, standardized data collection, quantitative parasite-density assessment, longer follow-up, and coverage of multiple transmission seasons would be valuable to confirm and extend these findings.

## Conclusion

This study demonstrates a predominance of *P. vivax* among adults presenting with malaria to two tertiary hospitals in Central Ethiopia, highlighting the continuing and potentially increasing importance of *P. vivax* in the local malaria burden. Both *P. vivax* and *P. falciparum* were associated with severe clinical presentations, with severe anaemia being the most frequently observed complication. However, no statistically significant association was observed between malaria species and WHO-defined severe malaria.

These findings underscore the importance of early diagnosis, accurate species identification, prompt and appropriate treatment, and careful clinical assessment for severe malaria in adults. Given the substantial burden of *P. vivax*, strengthening strategies for radical cure, including appropriate G6PD assessment and safe use of primaquine, remains important for reducing recurrent infection and supporting malaria control and elimination efforts. Further prospective, multicentre studies with longer follow-up and broader seasonal coverage are needed to better characterize determinants of severe malaria and the evolving epidemiology of *P. vivax* and *P. falciparum* in Central Ethiopia.

## Data Availability

The data supporting the findings of this study are openly available at the following link: 10.6084/m9.figshare.32301639.
